# Review on *D*-Allulose: *In vivo* Metabolism, Catalytic Mechanism, Engineering Strain Construction, Bio-Production Technology

**DOI:** 10.3389/fbioe.2020.00026

**Published:** 2020-02-03

**Authors:** Suwei Jiang, Wei Xiao, Xingxing Zhu, Peizhou Yang, Zhi Zheng, Shuhua Lu, Shaotong Jiang, Guochang Zhang, Jingjing Liu

**Affiliations:** ^1^Department of Biological, Food and Environment Engineering, Hefei University, Hefei, China; ^2^Anhui Key Laboratory of Intensive Processing of Agricultural Products, College of Food and Biological Engineering, Hefei University of Technology, Hefei, China; ^3^Carl R. Woese Institute for Genomic Biology, University of Illinois at Urbana–Champaign, Urbana, IL, United States

**Keywords:** *D*-allulose, *D*-allulose 3-epimerase, engineering strain, biological catalysis, *D*-tagatose 3-epimerase

## Abstract

Rare sugar *D*-allulose as a substitute sweetener is produced through the isomerization of *D*-fructose by *D*-tagatose 3-epimerases (DTEases) or *D*-allulose 3-epimerases (DAEases). *D*-Allulose is a kind of low energy monosaccharide sugar naturally existing in some fruits in very small quantities. *D*-Allulose not only possesses high value as a food ingredient and dietary supplement, but also exhibits a variety of physiological functions serving as improving insulin resistance, antioxidant enhancement, and hypoglycemic controls, and so forth. Thus, *D*-allulose has an important development value as an alternative to high-energy sugars. This review provided a systematic analysis of *D*-allulose characters, application, enzymatic characteristics and molecular modification, engineered strain construction, and processing technologies. The existing problems and its proposed solutions for *D*-allulose production are also discussed. More importantly, a green and recycling process technology for *D*-allulose production is proposed for low waste formation, low energy consumption, and high sugar yield.

## *D*-Allulose Physic-Chemical Characters and Application

*D*-Allulose has a molecular formula of C_6_H_12_O_6_ and a molecular weight of 180.16. The structural difference between *D*-allulose and *D*-fructose is located at the paired C_2_–C_3_ atoms (Chan et al., 2012). The physical characteristics of *D*-allulose include an appearance as white crystalline substance, odorless, low hygroscopicity, high solubility in water, and melting point of 96°C (Zhang et al., 2016a). *D*-Allulose is a kind of low energy monosaccharide sugar and present in very small quantities in natural products. And it is approximately 70% relative sweetness to sucrose with 0.2 Kcal/g that was 95% calorie reduction compared to sucrose and comprises only 0.3% of energy deposit in the body of animals (Chung et al., 2012).

The United States Food and Drug Administration (FDA) has confirmed the safety of *D*-allulose as generally regarded as safe (GRAS) food (Zhang et al., 2017). *D*-Allulose contains almost the same taste, performance, and texture with other sugars but it is mostly used by health concisions people globally for its very low energy. *D*-Allulose can be applied in food, pharmaceutical preparations, and dietary supplements (Granström et al., 2004). *D*-Allulose containing formulations were less prone to retrogradation in a starch-based composite gel matrix (Ilhan et al., 2020). Manufacturers widely add *D*-allulose in their food products in combination with other sweeteners. *D*-Allulose is globally applied in beverages (soft drinks and health drinks), savory dishes (soups, sauces, toppings, salads, and pickles), pharmaceuticals as a gelling agent, bakery products, ice cream, yogurt, and other low-calorie foods. Besides, *D*-allulose is also used as a thickening and stabilizing agent in bread, biscuits, rye cakes, and meat dishes.

*D*-Allulose exhibits almost zero calories with a low degree of energy density (Matsuo et al., 2002). *D*-Allulose can’t raise the blood sugar levels in diabetic patients and hence has been used to be a unique metabolic regulator of fat and glucose metabolism (Matsuo and Izumori, 2006, 2009; Hossain et al., 2011; Iida et al., 2013). Besides, *D*-allulose has also demonstrated a variety of activities, such as inhibitory activity toward intestinal digestive enzymes (Maeng et al., 2019), antioxidant enhancement (Sun et al., 2006), competitive transport through the intestinal mucosa with glucose (Kishida et al., 2019), enhancing glucokinase translocation of from the hepatic nucleus to cytoplasm (Shintani et al., 2017), strong anti-hyperlipidemic and anti-hyperglycemic effects (Hossain et al., 2015b), anti-inflammatory actions on adipocytes (Hossain et al., 2015a), preventing obesity and type 2 diabetes mellitus (Hossain et al., 2015b), and inhibiting trichomonad development (Harada et al., 2012). *D*-Allulose could be used as a health food or medicine for consumers with special needs. Thus, *D*-allulose has a potential application value as a substitute for glucose. Many health concisions individuals are shifting their preference toward consuming healthy food products. The application range of *D*-allulose is much broader than that of artificial non-sugar sweeteners (Yang et al., 2019). Hence, the global *D*-allulose market will observe robust growth over the forecast period.

## *D*-Allulose *In Vivo* Metabolism

*D*-Allulose belongs to a real and natural sugar rather than an artificial sweetener (Ran et al., 2019). After feeding, *D*-allulose hasn’t the same metabolic pathway as *D*-fructose in rats (Kishida et al., 2019). *D*-Allulose was highly stable in simulated gastric fluid, in fasted state simulated intestinal fluid and in human and rat hepatocytes, whereas *D*-fructose was rapidly metabolized (Maeng et al., 2019). Intestinal *D*-allulose transport was mediated by glucose transporter type 5 at lower affinity relative to *D*-fructose (Kishida et al., 2019). *D*-Allulose is not involved in glucose related metabolism (Iwasaki et al., 2018). Thus, *D*-allulose is not metabolized in an animal liver, and thus it is impossible to contribute to hepatic energy production (Maeng et al., 2019).

During the *in vivo* metabolism of *D*-allulose, a small quantity of *D*-allulose is decomposed into short-chain fatty acids under the action of cecal microbes (Kimoto-Nira et al., 2017). Most of *D*-allulose is excreted out of the body in the two forms of urine and feces after intake by oral administration or intravenous injection approaches. In the form of urine transported through the blood circulation, approximately 98% of *D*-allulose was excreted after intravenous injection (Whistler et al., 1974). *D*-Allulose is partly absorbed in the small intestine when orally administered in rats, and the remaining substance enters the blood circulation and is finally excreted out of the body via urine containing *D*-allulose (Iwasaki et al., 2018). Besides, in the form of feces, considerable *D*-allulose is excreted after the treatment of cecal microbes in the intestinum crassum of animals (Matsuo et al., 2003). In the *in vivo* metabolism of *D*-allulose, mice and dogs were previously used in animal experiments (Han et al., 2016; Nishii et al., 2016; Do et al., 2019). Human beings have a rich and developed sweat gland system that can discharge the liquid containing *D*-allulose in the body.

*D*-Allulose can’t be metabolized and *in vivo* converted into energy, but it plays an important role in the physiological activity of animals. *D*-Allulose was proved to have a significant anti-hyperglycemic effect and no abnormal clinical problems according to the result of the clinical study (Hayashi et al., 2010). Even the feeding amount reached 2 g *D*-allulose per Kg rat’s weight, no observed-adverse effect was shown based on *D*-allulose-associated reproductive toxicity (Kim et al., 2019). With dogs as the tested animals, all the dogs had a good appetite when 4 g/kg of *D*-allulose was administered, thus single oral dose of *D*-allulose didn’t show severe toxicity (Nishii et al., 2016). In addition, *D*-allulose could effectively decrease the activity of hepatic lipogenic enzymes and the levels of blood lipid and blood sugar (Chu et al., 2003; Kimura et al., 2005; Matsuo et al., 2015). The decreased hepatic lipogenic enzyme reduces the tissue weight of liver and abdominal adipose and inhibits fat accumulation (Chung et al., 2012). *D*-Allulose regulates the blood lipid by changing the activity of lipid-regulating enzymes (Han et al., 2016). Further, *D*-allulose could alter cholesterol metabolism by reducing serum PCSK9 levels (Kanasaki et al., 2019). All in all, the biological activities of *D*-allulose mainly focused on enhancing insulin tolerance (Shintani et al., 2017; Iwasaki et al., 2018), inhibiting postprandial blood glucose rise (Shintani et al., 2017), reducing abdominal fat accumulation (Do et al., 2019), and preventing diabetes (Fukada et al., 2010).

## The Enzyme for *D*-Allulose Production

### Sources of DTEase Family Enzymes

In 1994, Japanese scientist Ken Izumori discovered that *D*-tagatose 3-epimerase has the capability of converting *D*-fructose to *D*-allulose (Itoh et al., 1994). *D*-Tagatose-3-epimerases (DTEases) family is a kind of enzymes that catalyze the isomerization of C_3_ position of ketose monosaccharides and is also the core enzyme in the production of rare sugars (Jia et al., 2014). Normally, *D*-allulose is produced through the isomerization of *D*-fructose under the catalysis of DTEase family enzymes. DTEase family enzymes include DTEases (Yoshida et al., 2007; Zhang et al., 2009b), *D*-psicose 3-epimerases (DAEase) (Kim H.J. et al., 2006; Mu et al., 2013; Zhang et al., 2013a), and ketose 3-epimerase (Yoshida et al., 2019). All of these enzymes have the same characteristics that catalyze the conversion of *D*-fructose to *D*-allulose. The enzyme activity is generally calculated by measuring the amount of *D*-allulose with *D*-fructose as the catalysis substrate. One unit is defined as the amount of enzyme that catalyzes the production of 1 μmoL *D*-allulose per min at the optimal conditions (Zhu et al., 2019a).

DTEase from *Pseudomonas cichorii* ST-24 was initially identified in 1993 through hexulose catalytic epimerization (Ishida et al., 1997b). The genes coding for DTEase enzyme have been consecutively confirmed and isolated from *Agrobacterium tumefaciens* (Kim H.J. et al., 2006), *Rhodobacter sphaeroides* SK011 (Zhang et al., 2008), *Clostridium cellulolyticum* H10 (Mu et al., 2011), *Ruminococcus* sp. (Zhu et al., 2012), *Thermotoga maritima* (Sakuraba et al., 2009), and *Clostridium scindens* (Mu et al., 2013). The catalysis approaches of free enzyme and immobilization are used to convert *D*-fructose into *D*-allulose. Since DTEases were first discovered by Izumori and used to successfully catalyze the production of the rare sugars, the preparation of functional rare sugars by isomerases has become a research hotspot (Izumori and Okaya, 1993). At present, DTEases from only six strains have been identified as members of DTEases family enzymes. These strains contained *Pseudomonas cichorii* (Izumori and Okaya, 1993), *Agrobacterium tumefaciens* (Kim H.J. et al., 2006), *Rhodobacter sphaeroides* (Zhang et al., 2009b), *Clostridium cellulolyticum* (Mu et al., 2011), *Ruminococcus* sp. (Zhu et al., 2012), and *Desmospora* sp. (Zhang et al., 2013b). More DTEases sources and more efficient DTEases activity need to be further explored (Oh et al., 2007; Zhang et al., 2009a; Mu et al., 2011; Jia et al., 2014; Park et al., 2016a, b).

### Enzymatic Properties

DTEase family enzymes possess a highly conserved activity center and key amino acid residues with similar features, such as optimum temperature of 40°C–70°C, optimal pH of 7.5–9.0, molecular weights of 32–34 kDa for monomer, 64–68 kDa for dimer, 128–139 kDa for tetramer (Kim H.J. et al., 2006; Zhang et al., 2008, 2009b, 2013a; Mu et al., 2013). The half-lives of DTEase family enzymes vary from 15 min (Mu et al., 2013) to 408 min (Mu et al., 2011). The optimal equilibrium ratios (*D*-allulose:*D*-fructose) from *A. tumefaciens* DAEase and *P. cichorii* DTEase are 33:67 and 20:80, respectively (Ishida et al., 1997a; Kim H.J. et al., 2006) (Table 1).

**TABLE 1:** Properties of DTEase family enzymes.

	Optimum	Molecular	Optimal	Metal	Half-life^a^	Equilibrium	kcat/Km^c^
Sources of DTEase family enzymes	temperature	weights	pH	ions	(min)	ratio^b^	(min^–1^mM^–1^)
*Paenibacillus senegalensis* (Yang et al., 2019)	55°C	33.5	8.0	Mn^2+^	–	30:70	39
*Caballeronia fortuita* (Li et al., 2019)	65°C	33	7.5	Co^2+^	63	37.5:62.5	–
*Sinorhizobium* sp. (Zhu et al., 2019b)	50°C	–	8.0	Mn^2+^	–	–	–
*Staphylococcus aureus* (Zhu et al., 2019a)	70°C	134.13 (tetramer)	8.0	Mg^2+^	–	–	–
*Grobacterium* sp. ATCC 31749 (Tseng et al., 2018)	55–60°C	32	7.5–8.0	Co^2+^	267 (55°C)	30:70	–
*Arthrobacter globiformis* M30 (Yoshihara et al., 2017)	70°C	128 (tetramer)	7.0–8.0	Mn^2+^	–	–	–
*Flavonifractor plautii* (Park et al., 2016a)	65°C	33	7.0	Co^2+^	130	–	156
*Bacillus subtilis* (He et al., 2016)	55°C	–	7.5–8.0	–	170	–	–
*T. primitia* ZAS-1 (Zhang et al., 2016b)	70°C	–	8.0	Co^2+^	∼30	28:72(70°C)	144
*Dorea* sp. CAG317 (Zhang et al., 2015, 2018)	70°C	33	6.0	Co^2+^	∼30	30:70(70°C)	412
*C. bolteae* (Jia et al., 2014)	55°C	139 (tetramer)	7.0	Co^2+^	156	32:68(60°C)	107
*Clostridium* sp. (Mu et al., 2013)	65°C	130 (tetramer)	8.0	Co^2+^	15	28:72	141.4
*C. scindens* 35.704 (Zhang et al., 2013a)	60°C	132 (tetramer)	7.5	Mn^2+^	108	28:72(50°C)	64.5
*Ruminococcus* sp. (Zhu et al., 2012)	60°C	132 (tetramer)	7.5–8.0	Mn^2+^	96	28:72	51
*Clostridium cellulolyticum* (Mu et al., 2011)	55°C	132 (tetramer)	8.0	Co^2+^	408	32:68	186.4
*Rhodobacter sphaeroides* (Zhang et al., 2008)	60°C	64 (dimer)	9.0	Mn^2+^	–	23:77	–
*Agrobacterium Tumefaciens* (Kim H.J. et al., 2006)	50°C	132 (tetramer)	8.0	Mn^2+^	64	33:67	205
*Pseudomonas cichorii* (Itoh et al., 1994; Ishida et al., 1997a)	40°C, 60°C	68 (dimer)	7.5	–	–	20:80	–
*Bacillus subtilis* (Patel et al., 2019)	60°C, 70°C		6–11	Co^2+^ Mn^2+^		28:72	

*^*a,b,c*^Represent the optimum parameters of temperature, substrate, and pH, respectively. – Means not evaluated.*

Given their importance for *D*-allulose biotransformation, the kinetic parameters reflect the substrate affinity and catalytic efficiency of the enzyme. DAEase has the highest catalytic efficiency among the DTEase family enzymes. The kinetic parameters of *C. cellulolyticum* and *A. tumefaciens* DAEases were 186.4 and 205 mM min^–1^, respectively, which are higher than those from *Clostridium* sp. DTEase (141.4 mM min^–1^) and *Ruminococcus* sp. (51 mM min^–1^) (Mu et al., 2011, 2013; Zhu et al., 2012).

Metal ions play a pivotal role in the conversion of *D*-fructose into *D*-allulose by anchoring the bound of *D*-fructose. Kim et al. (2010) analyzed DAEase from *A. tumefaciens* for the conversion of *D*-fructose to *D*-allulose. The distance between *A. tumefaciens* DAEase residue Glu150 and Mn^2+^ is critical to the activity of DAEase (Kim et al., 2010). In addition, Asp183 and His209 residues bounded by metal ions are involved in efficient substrate binding. *C. cellulolyticum* H10 DAEase Glu150 and Glu244 perform the epimerization reaction of catalysis at the C_3_ position of *D*-fructose (Chan et al., 2012). The DTEase family enzymes have distinctly different degrees of dependency on metal ions of Mn^2+^, Co^2+^, and Mg^2+^ (Tseng et al., 2018; Yang et al., 2019; Zhu et al., 2019a). *A. tumefaciens* DAEase and *R. sphaeroides* DTEase activity could be remarkably enhanced when a metal ion Mn^2+^ is involved in the catalytic system (Kim H.J. et al., 2006; Zhang et al., 2008). *P. cichorii* DTEase does not require metal ions as a cofactor (Itoh et al., 1994; Ishida et al., 1997a). However, *C. cellulolyticum* DAEase and *C. scindens* DTEase are strictly Co^2+^ and Mn^2+^ metal-dependent (Mu et al., 2011; Zhang et al., 2013a). These two enzymes are completely inactivated in the absence of metal ions.

### Structural Features

The thermostability of recombinant enzymes has been ascribed to the high number of hydrophobic interactions and a more rigid configuration of their amino acid sequences (Polizzi et al., 2007). The DAEases from *A. tumefaciens* and *C. cellulolyticum* H10 exhibit the tightest tetramer formation among the DTEase family enzymes. In the tetramer of DAEase, the amino acid residues form 34 hydrogen bonds between the two subunits. The high catalytic activity of DAEase is attributed to the wide interface solvent-accessible areas, which are caused by the extensive interactions between the two dimers among the DTEase family enzymes (Yoshida et al., 2007; Sakuraba et al., 2009; Li et al., 2019). In addition, DAEase expressed by the recombinant strains still exhibited excellent enzymatic properties. The heterologous expression of *A. tumefaciens* and *C. cellulolyticum* H10 DAEase exhibits long half-life, high kinetic parameters, and high thermal stability (Kim H.J. et al., 2006; Mu et al., 2011).

The *A. tumefaciens* DAEase is a typical representative among the DTEase family enzymes. The tetrameric arrangement of *A. tumefaciens* DAEase is an asymmetric unit consisting of four identical subunits of A, B, C, and D (Yoshida et al., 2007; Chan et al., 2012). The active site contains a metal ion with four residues and octahedral coordination to two water molecules. These four residues are conserved among the DTEase family enzymes (Kim K.S. et al., 2006). These four subunits are the crystallographic symmetry-related dimers, in which subunit A and D interact and both are in close contact with subunit B and C. The active site of *A. tumefaciens* DAEase is exposed on the same front side of the dimers. These stable dimers provide a great accessible surface to bind the substrate on the front side of the dimers.

The hydrophobic groove of the active sites and the accessible surface is located in the middle of A and B subunits. The subunit sides of the DAEase are closed and exposed at the two ends of the barrel. In the tetramer of DAEase, two dimers are enclosed together at the closed sides of the barrel. The monomer (A, B, C, and D subunits) consists of eight repeat units of (β/α)_8_ structure. Each monomer is composed of 13 α-helices and 8 β-strands as the main structural motif. In addition, a (β/α)_8_ TIM barrel also exists in the active site of the monomer (Kim K.S. et al., 2006).

### Catalytic Mechanism

The catalysis of DTEase family enzymes depends on the molecular arrangement of each subunit. These subunits expose their active sites for the substrate to achieve efficient enzymatic reactions. Seven amino acid residues form hydrogen bonds with water molecules. The seven residues of *A. tumefaciens* DAEase/*P. cichorii* DTEase (Glu150/Glu152, Asp183/Asp185, His209/His211, Glu244/Glu246, Glu156/Glu158, His185/His188, and Arg215/Arg217) play an important role in substrate binding and thermal stability confirmed by site-directed mutagenesis (Yoshida et al., 2007). In addition, Trp112, Glu156, and Arg215 greatly influence the activity of the enzyme and the stabilization of *cis*-endiolate intermediate (Kim et al., 2010).

The epimerization in the active sites proceeds after the catalytic substrate displaces the water molecules. A proton from *D*-fructose C_3_ is removed to generate a *cis*-enediolate intermediate of *D*-allulose under the cooperation of two residues (Glu150 and Glu244) and Mn^2+^. In the residue sites of Glu150, Glu156, His209, and Glu244, the hydrogen bonds form between the *cis*-enediolate intermediate and *D*-allulose (Kim et al., 2010). *D*-Allulose is released from the position between the hydrogen bonds and water molecules in the active sites of DAEase.

Currently, the molecular modification has been performed to improve the catalytic activity and thermal stability of DTEase family enzymes. The site-directed mutagenesis was to improve the thermal stability and catalytic behavior of *L*-rhamnose isomerase from *Caldicellulosiruptor obsidiansis* for the production of *D*-allulose (Chen et al., 2018). The hydrophobic residues within β1-α1-loop were collectively replaced with polar amino acids. The V48N/G59N/I63N and V48N/G59N/I63N/F335S mutants respectively resulted in the increase of relative activities by 105.6 and 134.1% compared with that of the wild-type enzyme (Chen et al., 2018). Site-directed mutagenesis also improved the thermal stability of *D*-allulose 3-epimerase from *Dorea* sp. (Zhang et al., 2018). The *t*_1__/__2_ value of the mutant protein increased by 5.4-fold at 50°C and the Tm value increased by 17.54-fold compared with the wild-type enzyme (Zhang et al., 2018).

The recent study of the catalytic mechanism is still in the preliminary stage for DTEase family enzymes, and the relationship between enzyme structure and catalytic function needs to be deepened. Besides, some deficiencies still exist in thermal stability and substrate specificity for DTEase. The substrate specificity of DTEase family enzymes should be further utilized in the production of rare sugars to realize the efficient and green production of functional rare sugars.

## Construction of Engineered Strains For *D*-Allulose Bio-Production

Most of DTEase family enzymes have been identified and isolated from bacteria (Table 1). The amount of enzyme expressed in the native strains is far from the requirement of the application. Thus, constructing expression vector and expressing it in heterologous organisms are of great significance in characteristics investigation and enzymic application.

### *E. coli* Expression System

*Escherichia coli* expression system has the advantages of low cost and high expression efficiency. The soluble over-expression of DTEase family enzymes in *E. coli* was achieved and the recombined DTEase enzymes are separated and purified using affinity chromatography. The purified recombinant DTEase enzymes can be used to catalyze the production of *D*-allulose in a bioreactor via immobilization approach (Takeshita et al., 2000). The titer of *D*-allulose varies from 120 g L^–1^ to 218 g L^–1^ with a conversion yield of 24–33% (w/w) (Mu et al., 2011, 2013). With a *D*-fructose solution of 60% adjusted to pH = 7, approximately 25% of the substrate was converted into *D*-allulose (Takeshita et al., 2000). A concentration of 441 g L^–1^ *D*-allulose from 700 g L^–1^ *D*-fructose was produced with borate by immobilizing the recombinant DAEase onto duolite resins A568 and A7, whereas without borate, only 193 g L^–1^ *D*-allulose was produced (Lim et al., 2009). In addition, the production yield and conversion efficiency of recombinant *E. coli* cells expressing *Agrobacterium tumefaciens* DAEase are respectively 230 g L^–1^ and 33% (w/w) by using whole-cell reaction approach (Park et al., 2016b).

### *Bacillus* sp. Expression System

The recombinant *Bacillus subtilis* carrying DAEase from *Ruminococcus* sp. could overexpress DAEase with high efficiency and low-cost. The recombinant DAEase immobilized onto the anion exchange resin matrix could facilitate stable and effective *D*-allulose production. The activity of recombinant DAEase expressed by *B. subtilis* was 58.6 U/mg, which was higher than that in *E. coli* (8.95 U/mg) (Table 2). Besides, the expression regulatory element of DAEase also affects the amount and activity of the enzyme. Vector pMA5-P_xy/A_-RDPE could constitutively express 95 U/mL DAEase in *B. subtilis*. This value was higher than that of pBluescript II-SK-*DTE* expressed in *E. coli* (Ishida et al., 1997a; Chen et al., 2016). The activity of DAEase could be remarkably enhanced under the control of xylose-inducible promoter P_xy/A_. The enzymatic activity reached 95 U/mL in a 7.5 L fermentor through fed-batch fermentation (Chen et al., 2016).

**TABLE 2:** Bio-production of *D*-allulose by engineered strains.

	Enzyme and	Base	Host	Expression cassette	Activities, yields, and
Origin of gene	molecular weight	numbers	strains	and expression	publishing years
*P. cichorii* ST-24	DTEase, 32.5 kDa	873 bp	*E. coli* XLl-Blue	pBluescript II-SK-DTE soluble expression	0.35 U/mL (Ishida et al., 1997a), 1997
*C. scindens* ATCC 35704	DTEase, 31 kDa	864 bp	*E. coli* BL21	pET-22b (+)-DTE soluble expression	27. 9% (Xing et al., 2011), 2011
*C. cellulolyticum* H10 ATCC35319	DAEase, 32 kDa	882 bp	*E. coli* BL21	pET-22b(+)-Cc-*dpe*, soluble expression	218 g/L (Mu et al., 2011), 2011
*Ruminococcus* sp. 5_1_39BFAA	DAEase, 33 kDa	876 bp	*E. coli* BL21	pET-21a-*dpe*, soluble expression	8.95 U/mg, 125 g/L (Zhu et al., 2012), 2012
*Clostridium* sp. BNL1100	DAEase, 32 kDa	879 bp	*E. coli* BL21	pET-22b(+)-Clsp-*dpe*, soluble expression	120 g/L (Mu et al., 2013), 2013
*A. tumefaciens str.* C58	DAEase, 33 kDa	870 bp	*E. coli* BL21	pET22b (+)/*pelb*-*dpe*, secretion	10.9 U/mL, 179 g/L (Gu et al., 2013), 2013
*E. coli* JM109	DTEase, 29.8 kDa	789 bp	*E. coli* BL21	pET-15b-DTE, soluble expression	No data (He et al., 2015), 2015
*A. tumefaciens*	DAEase, 33 kDa	870 bp	*S. cerevisiae* AN120	pRS424-TEF_pr_-ss-xy/A soluble expression	12.0% (Li et al., 2015), 2015
*A. tumefaciens* ATCC 33970	DAEase, 33 kDa	870 bp	*E. coli* ER2566	pET-24a(+)-*dpe*, soluble expression	230 g/L (Park et al., 2016b), 2016
*Treponema primitia* ZAS-1	DAEase, 33.3 kDa	888 bp	*E. coli* BL21	pET-22b(+)-*dpe*, secretion	137.5 g/L (Zhang et al., 2016b), 2016
*Ruminococcus* sp. 5_1_39BFAA	DAEase, 33 kDa	876 bp	*B. subtilis* 1A751	pMA5-Pxy/A-RDPE, secretion	95 U/mL, 145 g/L (Chen et al., 2016), 2016
*Ruminococcus* sp. 5_1_39BFAA	DAEase, 33 kDa	876 bp	*B. subtilis*	pNCMO2-P2-*dpe*, soluble expression	58.6 U/mg (Li et al., 2018a), 2018
*A. tumefaciens*	DAEase, 33 kDa	870 bp	*K. marxianus*	pRS42H-*dpe*, soluble expression	190 g L^–1^ (Yang et al., 2018), 2018

### Yeast Expression System

The exogenous DAEase gene could be effectively expressed in the recombinant *S. cerevisiae* (Li et al., 2015) and *Kluyveromyces marxianus* (Yang et al., 2018). The expression vector of pRS424-TEF_pr_-ss-xy/A carrying *A. tumefaciens DAEase* could generate a 33 kDa protein in *S. cerevisiae* AN120 (Li et al., 2015). Both the xylose isomerase gene of *T. thermophilus* and *A. tumefaciens* DAEase gene co-expressed in yeast spores to strengthen the synergistic catalysis effect. These two recombinant enzymes were immobilized for *D*-allulose production with *D*-glucose as the substrate (Li et al., 2015). *D*-Fructose from *D*-glucose catalyzed by recombinant xylose isomerase is then converted to *D*-allulose by recombinant DAEase. Yang et al. (2018) have provided a valuable pathway to regenerate engineered *K. marxianus* cells, and thus achieve cyclic catalysis for *D*-allulose production. The recombinant *K. marxianus* produced 190 g L^–1^ *D*-allulose with 750 g L^–1^ *D*-fructose within 12 h. Approximately 100 g residual *D*-fructose was converted into 34 g ethanol by the engineering strains. Besides, the idea of cyclic catalysis for *D*-allulose production was also provided through a whole-cell reaction (Yang et al., 2018).

*Bacillus* sp., *E. coli*, and yeast are generally used to construct the recombinant system for DAEase expression. Different from *E. coli*, *B. subtilis* has no outer membrane, and the secreted protein can be released directly to the culture medium. The safety of *B. subtilis* is food grade. No heat source lipopolysaccharide (endotoxin) is mixed in the secreted protein products. Compared with *E. coli*, *B. subtilis* expression system is not perfect. The engineering *E. coli* has advantageous characteristics of clear genetic background, complete carrier receptor system, rapid growth, simple culture, and stable recombinants. Besides, the yeast expression system has its own characteristics of simple culture conditions, fast growth speed, high expression level, and simple operation. After translation, the protein can be processed and correctly modified. The deficiency of yeast expression system lies in low expression of cloned genes, long fermentation time, incorrect glycosylation of proteins and anti-cell division. Further, the high concentration of polysaccharide in supernatant is not conducive to the purification of recombinant protein.

Early researchers tend to use *E. coli* as host bacteria to study the expression and properties of DTEase family enzymes (Ishida et al., 1997a; Mu et al., 2011, 2013). Recently, many researchers have applied *B. subtilis* and yeast as host bacteria to express DTEase family enzymes for *D*-allulose production (Chen et al., 2016; Yang et al., 2018). The contents of *D*-allulose respectively reached 230 g/L (Park et al., 2016b) and 190 g L^–1^ (Yang et al., 2018) by using recombinant *A. tumefaciens* DAEase expressed in *E. coli* and *K. marxianus.* By the comparison, *E. coli* expression system of *A. tumefaciens* DAEase is the best among all the reported references shown in Table 2.

## Isolation and Purification of *D*-Allulose

Mass production of *D*-allulose is systematic engineering of gene acquisition, target gene expression, microbial fermentation, catalysis, *D*-allulose separation and purification. The researchers pay highly attention to the development of *D*-allulose with high efficiency and low cost. The following two methods are mainly used for the isolation and purification of *D*-allulose. The first method is ion exchange resin. Both anion exchange resin matrix and synchronous moving bed chromatography are used to immobilize DAEase for *D*-allulose production from *D*-fructose. The cells of *R. sphaeroides* SK011 produces 6.5 g L^–1^ *D*-allulose with a productivity of 0.82 g^–1^h^–1^ from initial 50 g L^–1^ *D*-fructose by using the dialysis method based on ion exchange resin (Zhang et al., 2009a). The toluene-treated *Sinorhizobium* sp. produces 37 g L^–1^ *D*-allulose from 700 g L^–1^ *D*-fructose; after the treatment of anion exchange resin matrix, the purity of *D*-allulose is up to 99.1%. Further, the purity of *D*-allulose reaches 98.3% via an approach of DTF-Ca^2+^ cation exchange chromatography (Xing et al., 2011). For the mixed system of *D*-allulose and *D*-fructose, *D*-fructose is first transformed into gluconic acid, and then *D*-allulose of 91.2% purity is obtained by anion exchange resin (Li et al., 2018b).

The second technique for *D*-allulose purification is the biological method. *D*-Allulose is obtained after the yeast consumed residual *D*-fructose to produce ethanol in the mixed system of *D*-allulose and *D*-fructose. The final purity of crystal *D*-allulose is 85% (Takeshita et al., 2000). Besides, the purity reaches approximately 86.2% by combining pervaporation technology, cation exchange chromatography, and biological method (Song et al., 2017). The biological method is more environment-friendly than ion exchange resin.

**FIGURE 1:**
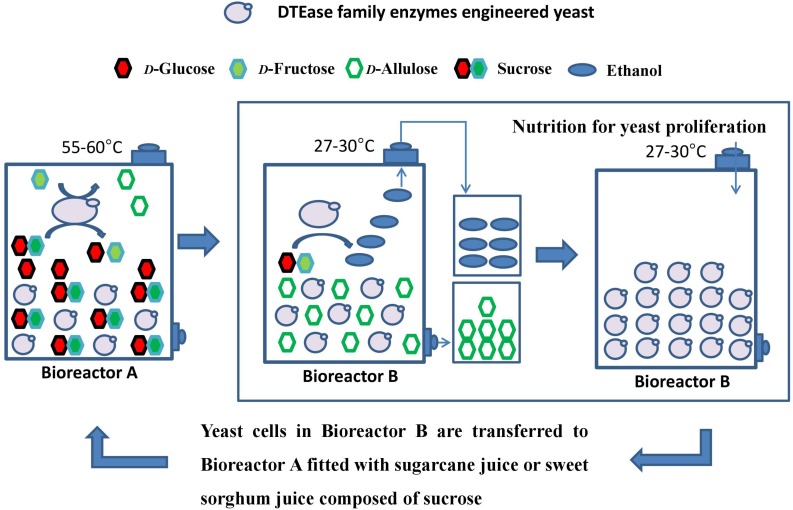
Recycling process for *D*-allulose conversion and ethanol production by using sugarcane juice or sweet sorghum juice as materials. During catalysis at a high temperature of 55–60°C for 1–2 h in Reaction A, most of the yeast can’t stand such high temperature and die. A small number of yeast spores still survive. These surviving yeast spores proliferate and consume *D*-fructose and *D*-glucose to produce ethanol at a later lower temperature of 27–30°C in Reaction B. Besides, a small part of *D*-fructose was metabolized by the remaining living yeast at such high temperature, but most of *D*-fructose was still converted into *D*-allulose in Reaction A.

A green and recycling process technology for *D*-allulose production is proposed in Figure 1. The whole reaction system is carried out between Bioreactor A (for sucrose hydrolysis and *D*-allulose conversion) and Bioreactor B (for ethanol production, *D*-allulose isolation, and yeast proliferation). Crude sugarcane juice or crude sweet sorghum juice are used as material to provide sucrose. Engineered yeast contains the native invertase and integrated exogenous DTEase family enzyme genes, thus engineered yeast is capable of sucrose hydrolysis to *D*-glucose and *D*-fructose by invertase, *D*-fructose conversion into *D*-allulose at 55–60°C, and ethanol production by fermenting *D*-glucose and *D*-fructose at 27–30°C. The advantages of this method include the low cost of raw materials, utilization of intermediate products produced in the process as much as possible, reduction of waste formation, reduction of energy consumption, and improvement of sugar yield.

## Prospects

The current industrial production of *D*-allulose has been achieved in China, Japan, South Korea, and United States. The production cost is still high due to the unsatisfactory activity of enzymes and their low reutilization frequencies. Therefore, increased enzymatic activities, stability, and catalytic frequency should be the key purposes in future research and development of DTEase family enzymes. Various advanced techniques and methods, such as directed evolution (Hong et al., 2016; Denisenko et al., 2017), screening of thermally stable enzymes (Baltaci et al., 2016), and material development of immobilized enzymes (Sahoo et al., 2016; Aldhrub et al., 2017), should be applied to modify DTEase family enzymes and thus overcome their unsatisfactory activity and stability.

The improvements of decolorization, desalination, crystallization, and drying reduce the production cost of *D*-allulose. The rational design or non-rational process with efficient high-throughput screening technology was the direct approach to modify the structure of DTEase family enzymes. The change of amino acid residues affects the advanced structure of DTEase, thus leading to the change of enzymatic properties. Site-directed mutagenesis was used to enhance the thermostability of DAEase from *R. baltica* (Mao et al., 2020) and improve the catalytic behavior of *C. obsidiansis L*-rhamnose isomerase (Chen et al., 2018). The improvement of DTEase family enzymes and production processing will decrease the production cost and *D*-allulose price, thus ensuring that *D*-allulose will be conveniently available to consumers.

## Author Contributions

SuJ performed the data analysis and revised the manuscript. WX wrote the manuscript. PY provided ideas. SL drew the pictures. ShJ drafted the work and provided the financial support. ZZ designed the manuscript. XZ, GZ, and JL searched the literature. All authors read and approved the final manuscript.

## Conflict of Interest

The authors declare that the research was conducted in the absence of any commercial or financial relationships that could be construed as a potential conflict of interest.
